# Histone H2A-Reactive B Cells Are Functionally Anergic in Healthy Mice With Potential to Provide Humoral Protection Against HIV-1

**DOI:** 10.3389/fimmu.2020.01565

**Published:** 2020-07-22

**Authors:** Amanda Agazio, Jennifer Cimons, Kristin M. Shotts, Kejun Guo, Mario L. Santiago, Roberta Pelanda, Raul M. Torres

**Affiliations:** ^1^Department of Immunology & Microbiology, University of Colorado, Aurora, CO, United States; ^2^Department of Medicine, Division of Infectious Diseases, School of Medicine, University of Colorado, Aurora, CO, United States

**Keywords:** B cells, peripheral tolerance, autoreactive, polyreactive, HIV-1, bnAbs, H2A

## Abstract

Peripheral tolerance is essential for silencing weakly autoreactive B cells that have escaped central tolerance, but it is unclear why these potentially pathogenic B cells are retained rather than being eliminated entirely. Release from peripheral tolerance restraint can occur under certain circumstances (i.e., strong TLR stimulus), that are present during infection. In this regard, we hypothesized that autoreactive B cells could function as a reserve population that can be activated to contribute to the humoral immune response, particularly with pathogens, such as HIV-1, that exploit immune tolerance to avoid host defense. In this study, we identify a population of autoreactive B cells with the potential to neutralize HIV-1 and experimentally release them from the functional restrictions of peripheral tolerance. We have previously identified murine monoclonal antibodies that displayed autoreactivity against histone H2A and neutralized HIV-1 *in vitro*. Here, we identify additional H2A-reactive IgM monoclonal antibodies and demonstrate that they are both autoreactive and polyreactive with self and foreign antigens and are able to neutralize multiple clades of tier 2 HIV-1. Flow cytometric analysis of H2A-reactive B cells in naïve wildtype mice revealed that these B cells are present in peripheral B cell populations and we further document that murine H2A-reactive B cells are restrained by peripheral tolerance mechanisms. Specifically, we show endogenous H2A-reactive B cells display increased expression of the inhibitory mediators CD5 and phosphatase and tensin homolog (PTEN) phosphatase and fail to mobilize calcium upon immunoreceptor stimulation; all characterized markers of anergy. Moreover, we show that toll-like receptor stimulation or provision of CD4 T cell help induces the *in vitro* production of H2A-reactive antibodies, breaking tolerance. Thus, we have identified a novel poly/autoreactive B cell population that has the potential to neutralize HIV-1 but is silenced by immune tolerance.

## Introduction

Immunological tolerance functions to remove or functionally silence autoreactive specificities from lymphocyte populations. Due to the stochastic nature of B cell receptor (BCR) rearrangement during development, 55–75% of early immature B cells possess an autoreactive BCR (1, 2). Receptor editing and clonal deletion reduce the frequency of autoreactive immature B cells in the bone marrow during central tolerance (3, 4). However, 20–35% of the B cells exiting the bone marrow maintain some level of autoreactivity (2, 5). These cells migrate to secondary lymphoid organs where they are rendered tolerant by mechanisms of peripheral tolerance including exclusion from the B cell follicles, lack of T cell help, and induction of a functionally inert state known as anergy (6–9). Although these autoreactive B cells are silenced by peripheral tolerance, the question remains—why retain a considerable frequency of potentially pathogenic B cells in the periphery? Of note, peripheral autoreactive B cells have been considered to contribute to the development of autoimmune disease in genetically predisposed individuals when mechanisms of peripheral tolerance fail (10–12). But do these autoreactive B cells serve a beneficial purpose in healthy individuals?

Molecular mimicry is a strategy employed by certain pathogens to subvert the host immune response and has often been implicated as an etiological basis for autoimmunity (13–17). By mimicking the sequence or structure of a host molecule, pathogens exploit immune tolerance and avoid detection. A relevant pathogen to study in the context of molecular mimicry and immune tolerance is the human immunodeficiency virus 1 (HIV-1). HIV-1 remains a global health burden and attempts at formulating a vaccine to eradicate this virus have thus far been unsuccessful. While the capacity of the virus to rapidly mutate clearly contributes to the poor immune response against HIV-1, it is now appreciated that immune tolerance also contributes to the ability of HIV-1 to evade immunity (18–24). The characterization of HIV-1 broadly neutralizing antibodies (bnAbs) isolated from infected HIV-1 individuals, now approaching approximately two hundred characterized bnAbs, has revealed that a large fraction of these bnAbs are also cross-reactive with autoantigens (25). HIV-1 appears to benefit from molecular mimicry, which is supported by studies that identified a portion of the conserved gp41 membrane proximal external region (MPER) on the HIV-1 envelope protein that mimics the sequence of the metabolic protein kynureninase (26). Furthermore, longitudinal studies of individuals infected with HIV-1 revealed that those who generate a neutralizing antibody response have immunological characteristics that suggest impaired immune tolerance such as fewer circulating T regulatory cells, increased circulating T follicular helper cells, and increased levels of serum autoantibodies (25, 27). Moreover, a recent report examining the B cell repertoires of HIV-infected individuals that either generate or do not generate bnAbs against HIV-1 indicates that the generation of HIV-1 bnAbs correlates with the presence of B cell lineages with antibody features associated with autoreactivity (28).

One consequence of B cell tolerance is the reduction of B cell diversity in the primary B cell repertoire, which could potentially limit the detection of foreign antigens. Therefore, we reasoned that autoreactive B cells may be temporarily retained in the periphery to protect against pathogens that utilize molecular mimicry to evade the host response. Indeed, autoreactive B cells are able to contribute to protective humoral immunity, and in doing so, reduce their autoreactivity via somatic hypermutation while increasing their affinity for the foreign antigen (29–31). The goal of this study was to identify a population of autoreactive B cells with the potential to neutralize HIV-1 and induce a breach in tolerance to allow them to contribute to a protective response.

Mice are not infected by HIV-1. Nevertheless, they can be immunized with HIV-1 antigens to investigate how to elicit a virus-specific and neutralizing antibody response. Our lab has previously demonstrated that wild type mice in which peripheral tolerance is either experimentally or genetically impaired have the ability to generate neutralizing HIV-1 antibodies that display limited breadth (23). Moreover, the ability to neutralize HIV-1 correlated with increased levels of serum IgM autoantibodies against histone H2A and monoclonal H2A IgM antibodies generated from immunized autoimmune-prone mice neutralized tier 2 HIV-1 clade B variants (23). The study described herein extend our previous findings to identify properties of H2A-reactive antibodies associated with HIV-1 neutralization and to characterize the tolerance status and response abilities of the B cells that produce them. Specifically, we have characterized additional H2A-reactive monoclonal antibodies (mAbs) with regard to *Ighv* gene usage, somatic mutation, poly/autoreactivity, and ability to neutralize HIV-1. We have further identified H2A-reactive B cells in wild type mice and show that these autoreactive mouse B cells are functionally anergic. Importantly, we also demonstrate that anti-H2A specific IgM and IgG can be elicited by these B cells when stimulated with toll-like receptor (TLR) agonists in the presence of either help from T cells of autoimmune-prone mice or artificial simulation of CD40 signaling. Together, our data show that immune tolerance silences a novel autoreactive B cell population that express antigen receptors able to cross-reacts with HIV-1, and that these B cells can be activated to produce antibody under certain circumstances. Altogether these results have implications for understanding the biology of autoreactive B cells and how to harness their specificity for use in a protective antibody response.

## Materials and Methods

### Mice

Wild-type C57BL/6J, B6.Sle123 (B6.NZM-Sle1/ Sle2/Sle3NZM2410/Aeg/LmoJ), and Sle1yaa (B6.Cg-Sle1NZM2410/Aeg Yaa/DcrJ) were purchased from The Jackson Laboratory and bred in specific pathogen–free conditions in the animal facility at the University of Colorado-Anschutz Medical Campus (Aurora, CO). MD4 BCR Transgenic (C57BL/6-Tg(IghelMD4)4Ccg/J) mice and μMT B cell deficient (B6.129S2-Ighmtm1Cgn/J) mice were purchased from Jackson Laboratory and bred to generate MD4 μMT mice. Male and female mice were used between 8 and 12 wk (young) and 30–52 (old) wk. All experiments were approved and performed in accordance with the University of Colorado Anschutz Medical Campus Animal Care and Use Committee.

### Hybridoma Generation and IgM Purification

Pristane treated C57BL/6 mice were treated with 500 μl pristane oil, then immunized with gp140 envelope and Alu-gel-s (alum) 30 days later (32). Splenocytes from two pristane treated mice immunized 14 days previously with gp140 and that displayed HIV-1 neutralization with serum antibodies (Figure S1) were fused with SP2 myeloma cells using polyethylene glycol, diluted into 96-well plates, then treated with selection media (0.5 μg/ml azaserine and 14 μg/ml hypoxanthine) to eliminate unfused SP2 cells. Remaining clones were then tested for H2A-reactivity using ELISA and 1% bovine serum albumin as a blocking reagent. Positive clones were selected and expanded, then retested for H2A-reactivity with 1% type A gelatin from porcine skin blocking reagent to eliminate false positive clones. Remaining positive clones were expanded into T175 flasks and supernatant was collected for antibody purification. IgM was purified using an affinity purification column in which anti-mouse IgM (rat IgG2a; clone R33-24.12) (33) was covalently bound to Sepharose beads. IgM was eluted from the column using 0.1 M glycine HCl pH 2.8 buffer, then buffer exchanged into PBS using 100 kD cutoff centrifugal filter (Millipore). Twelve H2A-reactive clones were identified from sixteen 96-well plates, and three clones showed robust reactivity against H2A following purification. Hybridomas were also generated from LPS anti-CD40 treated splenocytes. In brief, a spleen from one mouse was prepared into a single cell suspension and treated with 20 ng/ml BAFF (R&D Systems), 20 μg/ml LPS (Sigma-Aldrich), and 10 μg/ml anti-CD40 (FGK.45, made in house) for 2 days. Splenocytes were then fused with myeloma cells (SP2), treated with selection media, and screened for H2A-reactivity as previously described. Sixteen H2A-reactive clones were identified from eight 96-well plates, and six clones showed robust reactivity against H2A following purification.

### Production and Purification of HIV Envelope Protein

Trimeric gp140 (YU2) was produced as previously described (34). In brief gp140 was generated by transient transfection of COS7 cells (ATCC) using 5 μg of gp140 plasmid (gift from T.M. Ross, University of Georgia, Athens, GA) and Escort IV Transfection Reagent (Sigma-Aldrich). Purification of gp140 was achieved using a column made with agarose-bound *Galanthus nivalis* lectin (Vector Laboratories). Gp140 was bound to the column, washed with PBS, and eluted using 1M methyl mannopyranoside (Sigma-Aldrich). The purified protein in eluant was buffer exchanged into sterile PBS and concentrated using Vivaspin 20 centrifugal filters with a 30-kD cutoff (Sartorius). Protein purity was checked by SDS-PAGE. Purified gp140 was stored at −20°C and aliquoted to prevent multiple freeze/thaw cycles.

### Foreign and Autoantigen ELISAs

To detect Ig reactive against various antigens, 96-well Nunc-Immuno MaxiSorp plates (Thermo Fisher Scientific) were coated with antigen diluted into PBS and incubated overnight at 4°C. Concentrations for various coating antigens; 1 μg/mL of H2A or H2B (New England Biosciences), 10 μg/ml of chromatin from calf thymus (Sigma-Aldrich), 1 μg/ml of Smith antigen (Meridian Life Sciences), 1 μg/ml of myeloperoxidase (EMD Millipore), 5 μg/ml of insulin (Sigma-Aldrich), 2 μg/ml of ovalbumin (Biosearch Technologies), 1 μg/ml of gp140, 10 μg/ml of lipopolysaccharides from *Escherichia coli* O26:B6 (Sigma-Aldrich), 1 μg/ml of *Toxoplasma gondii* P32 (MyBioSource), 1 μg/ml of Influenza A H3N2 hemagglutinin (Novus Biologicals), 10 μg/ml of Pneumococcal Polysaccharide Powder Type 22F (ATCC), or 1 μg/ml of the RSC3 CD4 binding site protein (National Institutes of Health AIDS Reagent Program). For cardiolipin, plates were coated with 50 μg/ml cardiolipin sodium salt (Sigma-Aldrich) diluted in 100% molecular grade ethanol (Thermo Fisher Scientific) and allowed to evaporate completely overnight at 4°C. Plates were then washed with 0.5% Tween-20 in PBS and blocked using 1% BSA, 1 mM EDTA, 0.05% NaN_3_ blocking buffer for 2 h at 37°C. Serum samples were diluted in blocking buffer 1:20, loaded into plate, serially diluted 1:3 for a total of eight dilutions, and incubated overnight at 4°C. For purified hybridoma antibodies, samples were added to the plate at a concentration of 10 μg/ml, serially diluted 1:3 for a total of eight dilutions into blocking buffer, and incubated overnight at 4°C. Plates were washed then incubated with isotype specific (IgM or IgG) goat anti-mouse alkaline phosphatase (AP) conjugated antibodies (SouthernBiotec) at a dilution of 1:2,000 in blocking buffer for 1 h at 37°C. Plates were washed and then developed by adding 1 mg/ml of 4-nitrophenyl phosphate disodium salt hexahydrate (Alkaline Phosphatase Substrate; Sigma-Aldrich) diluted in developing buffer (1M diethanolamine, 8.4 mM MgCl2, and 0.02% NaN3, pH 9.8). Absorbance values were read at 405 nm on VersaMax ELISA reader (MDS Analytical Technologies). Titers of autoantigen reactive IgM antibodies were calculated in GraphPad Prism 7 by log transforming the reciprocal dilution values and calculating EC50 values using the sigmodal dose response with variable slope equation. EC50 values were then normalized to EC50 values calculated from B6Sle123 serum sample positive control. For autoantigen reactive serum IgG, raw optical density values were normalized to optical density obtained from B6.Sle123 serum positive control.

### HIV-1 Production and Viral Titration

Pseudoviruses Bal.26, SF162LS, 398F1, 25710, and X1632, and full-length virus YU2, were obtained through the National Institutes of Health AIDS Reagent Program, Division of AIDS, NIA ID, NIH. The JR-FL plasmid was a gift from P. Clapham at the University of Massachusetts. Viruses were produced using transient co-transfection of 5 μg/ml of gp160 encoding plasmid and 10 μg/ml of SG3Δenv plasmid in 293T cells as previously described (23). In short, 293T cells were transfected with plasmids using calcium chloride for 16 h and allowed to grow for an additional 24 h. Viral supernatants were collected and overlaid on top of a 20% sucrose cushion, then ultra-centrifuged for 2 h at 100,000 × g at 4°C. RPMI was added to viral pellets and incubated overnight to allow for resuspension. Viral infectivity was standardized in TZMbl cells (35), in which virus stocks were diluted starting at 1:100 in DMEM and serially diluted 1:3 for a total of eight dilutions in total volume of 100 μl in white opaque, 96-well CulturPlates (Perkin Elmer). TZMbl cells were infected by adding 10,000 cells to each well in 100 μl of DMEM with glutamax, pen-strep, and 10% FBS. Cells were lysed 48 h later by addition of luciferase substrate (Britelite plus Reporter Gene Assay system; Perkin Elmer). Plates were read on a Victor X light luminescence plate reader (Perkin Elmer) following a 2-min incubation with the luciferase substrate. Relative light units (RLU) were plotted against reciprocal dilutions to calculate the dilution of virus needed to produce a mean of 150,000 RLUs. These titers were then used to infect cells in the TZMbl neutralization assays.

### TZMbl Neutralization Assays

TZMbl neutralization assays with mouse serum and purified antibodies were performed as previously described (23, 35) and as specified by the Montefiori website (Duke University). The National Institutes of Health AIDS Reagent Program provided the TZMbl cells. Experiments were performed in BSL2+ and BSL3 facilities in accordance with UCD Biosafety protocols. For neutralization assays using mouse serum, all samples were heat inactivated at 55°C for 45 min prior to assaying. Serum samples were then diluted at 1:15 then serially diluted 1:3 for a total of eight dilutions into DMEM for a total volume of 100 μl. For purified monoclonal antibodies, samples were diluted to a concentration of 50 μg/ml then serially diluted 1:3 for a total of 8 dilutions into DMEM with final volume of 100 μl. Each HIV-1 strain was prepared at a viral titer that was previously determined to give a standardized infection measured by 150,000 RLUs (see previous section). HIV-1 strains and sample dilutions were then combined at 1:1 ratio, 50 μl of each preparation, and incubated for 2 h at 37°C, 5% CO_2_ to allow for antibody neutralization. TZMbl cells were then added, with a final count of 10,000 cells per well in 100 μl of complete DMEM + 10% FBS. Cell only controls and virus controls were included on each plate. Cells were then incubated for 48 h at 37°C, 5% CO_2_. To obtain results from each plate, 100 μl of supernatant was removed from each well, replaced with 100 μl of luciferase substrate, and pipetted up and down to lyse the TZMbl cells. Results were read as described in the previous section. To validate the neutralization of each HIV-1 strain, 2 μg/ml of VRC01 monoclonal antibody was included as a positive control for all neutralization assays. Serum and purified antibody neutralization values were expressed as the reciprocal of the serum dilution that produces 50% neutralization, or the inhibitory dose 50 (ID50). An Excel macro provided by D. Montefiori (Duke University) was used to calculate the percent neutralization.

### *Ighv* Gene Cloning and Analysis

RNA was isolated from hybridoma cell lines according to manufacturer's protocol using TRIzol (Invitrogen). Contaminating DNA was then removed from RNA samples using Ambion DNA-free kit (Ambion). cDNA was generated using SuperScript IV Reverse Transcriptase (Invitrogen) and Oligo(dT) primers (Invitrogen) according to manufacturer's protocol. Igh genes were amplified by PCR with the Platinum Taq DNA Polymerase High Fidelity kit (Invitrogen) using a degenerate forward VH primer and a mixture of reverse primers for JH segment primers as previously described (36). PCR products were purified by gel-electrophoresis and extracted using the QIAquick gel extraction kit (Qiagen). Igh PCR products were expanded using TOPO cloning (Invitrogen) and several clones from each hybridoma amplification were submitted for sequencing (Eton Bioscience Inc.) to ensure hybridoma cell lines were monoclonal. Igh gene segments were identified using the NCBI IgBlast tool.

### Immunizations and Sera Collection

For HIV envelope immunizations, doses of 50 μg of gp140 per mouse were diluted in sterile PBS and mixed 1:1 with Alu-Gel-S (Thermo Fisher Scientific) as an alum adjuvant. Immunizations with recombinant human histone H2A protein (New England BioLabs) were also given in 50 μg doses mixed 1:1 with Alu-Gel-S. All preparations of immunogens in Alu-Gel-S were incubated for at least 4 h rotating at 4°C prior to administration. Preparations of H2A conjugated to sheep red blood cells (SRBC) were generated as previously described (30). In brief, 8 × 10^9^ SRBC (Innovative Research) were washed three times in 30 ml cold PBS and resuspended in 1 ml cold conjugation buffer (0.35 M D-Mannitol and 0.01 M sodium chloride in distilled water). Then, 100 μg of H2A was added to the resuspension. This mixture was incubated at 4°C rocking for 10 min before adding 100 μl of 100 mg/ml N-(3- Dimethylaminopropyl)-N-ethylcarbodimide hydrochloride solution (Sigma-Aldrich) and incubating for an additional 30 min rocking at 4°C. The conjugated SRBCs were washed four times with 30 ml of cold PBS. The conjugation of H2A to the SRBC was confirmed by flow cytometry using a polyclonal rabbit anti-human histone H2A IgG (Novus Biologicals) and secondary goat anti-rabbit IgG-FITC (Southern Biotech) compared to rabbit IgG isotype control (SouthernBiotec). Mice were injected intravenously (i.v.) with 2 × 10^8^ H2A-SRBCs. For serum collection, blood was collected from mice by cheek puncture using GoldenRod 4 mm lancets (Fisher Scientific) into Z-gel serum micro tubes (Sarstedt). Blood was allowed to coagulate for 45 min at room temperature, then centrifuged at 10,000 × g for 5 min. Serum was then collected and stored at −20°C.

### Hemagglutination Assay

Serum samples were diluted 1:10 then serially diluted 1:2 down the rows of a V-bottom 96-well plate (Corning; Fisher Scientific). SRBCs were washed three times in cold PBS, then diluted to obtain a 0.5% SRBC solution in PBS, and 50 μl of this solution was added to each well containing a serum dilution. Samples were incubated for 1 h at 37°C. The highest dilution at which the serum agglutinated SRBCs was reported as the titer.

### Splenocyte Preparation

Single-cell suspension of splenocytes were obtained by mechanical digestion through a 100 μm nylon cell strainer (Celltreat) into 5 ml of DMEM + 5% FBS. To remove erythrocytes and decrease the amount of apoptotic cells in cell preparations, 5 ml of splenocyte suspensions were then carefully added on top of 9 ml of Ficoll-PAQUE Premium (GE Healthcare), and centrifuged for at 2,300 RPM for 20 min at room temperature with no centrifuge brake. The leukocyte containing buffy coat layer was then collected and washed three times in cold DMEM + 5% FBS. Cell counts were performed using hemacytometer or automated Vi-CELL Cell Counter (Beckman Coulter).

### H2A-Reactive B Cell Enrichment and Flow Cytometry

Human recombinant H2A (New England Biosciences) was conjugated to biotin using the EZ-Link Sulfo-NHS-LC-Biotin conjugation kit (Thermo Scientific) according to manufacturer's protocol. Antigen specific, H2A-reactive B cells were enriched as previously described (10, 37). In brief whole splenocytes from one mouse per sample were stained with 10 μg/ml of H2A-biotin in 250 μl PBS, 1% BSA, 1 mM EDTA, 0.05% NaN3 (FACs buffer) for 15 min at 4°C. Cells were washed twice, then fixed using 2% paraformaldehyde in PBS for 10 min 4°C. Cells were washed twice then stained with streptavidin-Alexa Fluor 647 (BioLegend) for 15 min at 4°C. Cells were washed twice, then stained with Anti-Cy5/Anti-Alexa Fluor 647 MicroBeads (MACS Miltenyi Biotec) for 15 min 4°C according to manufacturer's protocol. Cells were washed then applied to magnetic LS columns (MACS Miltenyi Biotec), and H2A-reactive positive fractions were collected. All samples were treated with 2.4G2 (made in house) to block nonspecific antibody binding to Fc receptors. H2A-reactive cells were then stained with for live/dead exclusion using Ghost dyes Violet 450 or Red 780 (Tonbo Biosciences), and/or 7AAD (eBioscience) when live cell preparations were used. H2A-reactive cells were also stained for the following cell surface markers; CD19-BV510 (Clone ID3; BDBiosciences), B220-BV605 (Clone RA3-62B; BioLegend), IgM-PeCy7 (Clone II/41; eBioscience), CD21-FITC (Clone 7E9; BioLegend), CD23-BV786 (Clone B3B4; BDBiosciences), CD1d-PE (Clone 1B1; eBioscience), CD24-PerCP-Cy5.5 (Clone M1/69; eBiosciences), and CD5-BV510 (Clone 53-7.3; BioLegend). For intracellular PTEN staining, fixed cells were permeabilized and stained in fresh 0.5% Saponin in FACs buffer with anti-PTEN-PE (Clone A2B1; BDBiosciences), or isotype control PE Mouse IgG1, κ (Clone MPOC-21; BDBiosciences). Analysis of apoptotic H2A-reactive B cells was performed using H2A directly conjugated to DyLight 650, and staining with 7AAD and ghost dye. Data was collected on the BD LSRFortessa X-20 Cytometer (BDBiosciences) and analyzed in FlowJo 10.

### Calcium Mobilization Assays

Freshly isolated splenocytes were stained with H2A-biotin, 7AAD (eBioscience), polyclonal goat anti-mouse IgM Fab-FITC (Jackson ImmunoResearch), CD23-PE (Clone B3B4; BioLegend), B220-PeCy7 (Clone RA3-62B; BioLegend), and CD24 APC-eFluor 780 (Clone M1/69; eBioscience) in DMEM + 2% FBS for 15 min at room temperature. Cells were then washed and stained with anti-biotin Fab APC (gift from J. Cambier, University of Colorado) to visualize H2A-reactive cells without crosslinking the BCR. Cells were washed and resuspended in DMEM + 2% FBS with 1 μmol/L Indo 1-AM (eBioscience), then incubated for 1 h at room temperature. Cells were washed and resuspended at a concentration of 10 × 10^6^ cells/ml in warmed DMEM + 2% FBS and placed on the BD LSRFortessa X-20 Cytometer to record 30 s of baseline readings before stimulation with 10 μg/ml of polyclonal F(ab′)2 goat anti-mouse IgM (Jackson ImmunoResearch). For H2A stimulated controls, H2A was directly conjugated to Dylight 650 using the Dylight 650 Pierce labeling kit (Thermo Fisher Scientific), and cells were directly stimulated with 10 μg/ml of H2A-Dylight 650. Kinetic analyses were performed using FlowJo 8 software.

### *In vitro* Toll-Like Receptor B Cell Stimulations

Untouched B cells were purified from C57BL/6 spleens using negative selection with CD43 MicroBeads (MACS Miltenyi Biotec). Purified B cells were added 2 × 10^6^ cells/ml to 24-well plates with a final volume of 1 ml. Cells were cultured in complete DMEM + 10% FBS with 20 ng/ml recombinant mouse BAFF (R&D Systems) in media alone or treated with 10 μg/ml of either Poly(I:C) (Invivogen), *Escherichia coli* lipopolysaccharide O26:B6 (Sigma-Aldrich), R848 (Resiquimod; Invivogen), or Class B CpG ODN 1826 (Invivogen). Culture supernatants were collected after incubation for 10 days and analyzed by ELISA for total IgM concentration, H2A-reactive IgM, Gp140-reactive IgM, and Chromatin-reactive IgM. For ELISAs with *in vitro* samples, plates were coated with 5 μg/ml of H2A or gp140, or 10 μg/ml of chromatin in PBS. Undiluted culture supernatants were added to each well, and ELISAs were completed as previously described. Data for antigen-reactive IgM is represented as 405 nm optical density values normalized to total IgM 405 nm optical density.

### *In vitro* T Cell B Cell Co-cultures

Untouched T cells were purified from C57BL/6 or B6Sle123 spleens using the CD4 T cell isolation kit (MACS Miltenyi Biotec), and stimulated for 24 h using 2 μg/ml of anti-CD28 (clone 3751; made in house), 20 U/ml of recombinant human IL-2 (Peppro Tech), and plate bound 0.5 μg/ml of anti-CD3 (clone 1452C11; made in house). Purified B cells from C57BL/6 spleens were isolated as described in previous section and combined with purified T cells in 24-well plates. Each well contained 1.5 × 10^6^ B cells and 1.5 × 10^6^ T cells in a final volume of 2 ml. Each cell type was also plated alone for controls. For transwell cultures, 1.5 × 10^6^ B cells were added to the bottom of a 24-well plate, and 1.5 × 10^6^ T cells were added to the top of a Corning 0.4 μm transwell chamber (Sigma-Aldrich) with a final volume of 2 ml. Following incubation for 8 days, culture supernatants were then collected and analyzed using ELISA as described in previous sections. Data represented as 405 nm optical densities for antigen-reactive IgM normalized to 405 nm optical density values from culture supernatants collected from B cells alone.

### *In vivo* Toll-Like Receptor and Anti-CD40 Stimulations

Serum was collected from young C57BL/6 mice prior to treatment to obtain baseline antibody levels. Mice were then treated with PBS and 50 μg rat IgG2a isotype control (Clone 2A3; BioXcell), or 50 μg rat anti-mouse CD40 (Clone FGK45; made in house) with either 100 μg of Poly(I:C) (Invivogen), 50 μg of *Escherichia coli* lipopolysaccharide O26:B6 (Sigma-Aldrich), 100 μg R848 (Resiquimod) (Invivogen), or 100 μg Class B CpG ODN 1826 (Invivogen). Serum was collected 7 days after treatment. Baseline and day 7 serum was then analyzed by ELISA as described in previous sections.

### Statistics

GraphPad Prism (v7) was used to calculate statistics. Statistical significance was calculated using a two-tailed Student's *t*-test for comparison between two groups. For multiple groups or groups with varying timepoints and/or conditions a one-way ANOVA or two-way ANOVA with Tukey's *post hoc* test was used, respectively. A *P*-value ≤ 0.05 was denoted as ^*^ and considered to be significant followed by ^**^P < 0.001; ^***^P < 0.0001; and ^****^P < 0.00001. If statistics were absent, data was not statistically significant (*P* > 0.06). Data were graphed as the mean ± SD.

## Results

### H2A-Reactive Monoclonal Antibodies From Autoimmune Mice Are Often Autoreactive and Polyreactive

We previously found that in response to gp140 (YU2) immunization of B6.Sle123 autoimmune-prone mice, or wild type mice after an experimental breach of peripheral tolerance, neutralizing serum antibodies against tier 2 HIV-1 variants could be elicited (23). Importantly, in both immunized autoimmune mouse strains, neutralizing serum activity correlated with elevated IgM anti-H2A serum titers and isolated H2A-reactive IgM mAbs derived from immunized B6.Sle123 mice displayed HIV-1 neutralizing activity (23).

**Figure 1:**
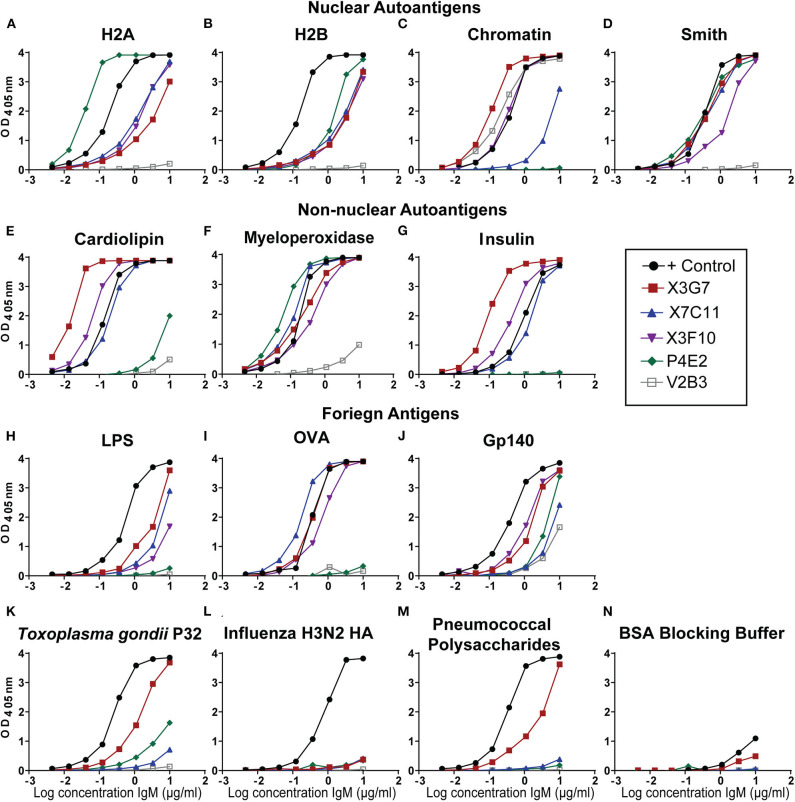
H2A-reactive monoclonal antibodies from autoimmune wild type mice recognize multiple self and foreign antigens. Purified IgM mAbs from the indicated H2A-reactive hybridomas were tested or reactivity against nuclear autoantigens, **(A)** histone H2A, **(B)** histone H2B, **(C)** Chromatin, **(D)** Smith, non-nuclear autoantigens **(E)** Cardiolipin, **(F)** Myeloperoxidase, **(G)** Insulin, and foreign antigens **(H)** Ovalbumin, **(I)** Trimeric HIV envelope protein gp140 (YU2), **(J)** Lipopolysaccharide, **(K)** the P32 antigen from *Toxoplasma gondii* **(L)** Influenza hemagglutinin from the H3N2 strain, **(M)** the 22F mixture of pneumococcal polysaccharides, and **(N)** 1% bovine serum albumin ELISA blocking buffer.

To determine if H2A-reactivity was relevant for HIV-1 neutralization in an additional autoimmune-prone mouse model, particularly of induced autoimmunity, we treated C57BL/6 mice with the hydrocarbon oil, pristane. Pristane emulsification is often used as an adjuvant for immunization, however; large doses (500 μL) of pristane reproducibly induces autoantibodies in all strains of mice (32, 38). Specifically, 30 days after intraperitoneal injection of pristane, wild type mice develop an SLE-like disease characterized by a strong interferon-alpha signature, production of anti-nuclear antibodies, and lupus nephritis (38, 39). Here, we immunized pristane-treated mice with gp140 and harvested spleens and collected serum on day 14. As previously shown (23), serum from pristane-treated and gp140 immunized mice harbored antibodies able to neutralize tier 2 HIV-1 variants (Figure S1). Splenocytes from mice harboring serum antibodies able to neutralize HIV-1 (Figure S1) were used to generate hybridomas, which were screened for H2A-reactivity. Twelve H2A-reactive clones were identified from sixteen 96-well plates, three of which displayed robust reactivity against H2A following purification and analysis at a standard concentration. Figure 1 shows that mAbs from three of the hybridomas displayed robust H2A-reactivity following purification (clones X3G7, X7C11, and X3F10). Clone V2B3, which does not react with H2A but does react with chromatin was also isolated from a pristane treated gp140 immunized mouse and included in analyses as a negative control (Figures 1A,C).

Many of the HIV-1 bnAbs identified in humans are polyreactive and, specifically, not only recognize a neutralizing gp120 epitope but also display autoreactivity. As such, we evaluated our H2A-reactive IgM mAbs for poly/autoreactivity. Purified mAbs were tested by ELISA against a panel of various self and foreign antigens. Serum from an autoimmune B6.Sle123 mouse was used as a positive control. We found that all three of our H2A-reactive monoclonal antibodies cross-reacted with a panel of nuclear autoantigens (Figures 1A–D) and non-nuclear autoantigens (Figures 1E–G). We further tested our H2A-reactive mAbs against a panel of foreign antigens comprised of chicken ovalbumin (OVA), HIV-1 gp140, lipopolysaccharide (LPS), *Toxoplasma gondii* P32 antigen, H3N2 influenza hemagglutinin antigen, and a cocktail of pneumococcal polysaccharides. These results revealed that the X3G7, X7C11, and X3F10 anti-H2A mAbs that displayed polyreactivity against multiple autoantigens and also cross-reacted with several of the foreign antigens tested (Figures 1H–M). Interestingly, clone X3G7 displayed the most polyreactivity as it was the only H2A-reactive mAb that also recognized the P32 antigen from *Toxoplasma gondii* and pneumococcal polysaccharides (Figures 1K,M). Importantly, none of the mAbs tested reacted with the bovine serum albumin (BSA) which was used as our blocking reagent (Figure 1N). Together these results demonstrate that the three H2A-reactive mAbs generated from pristane-treated and gp140 immunized mice also display autoreactivity toward other self-antigens and are also polyreactive against certain foreign antigens, including HIV-1 gp140. The recognition of foreign antigens by the H2A reactive mAbs suggests that these specificities may have the capacity to participate in a protective response against a foreign antigen or pathogen.

### H2A Monoclonal Antibodies That Neutralize HIV-1 Lack Somatic Hypermutation and Use Immunoglobulin Heavy Chain Genes Similar to Human HIV-1 bnAbs

**Figure 2:**
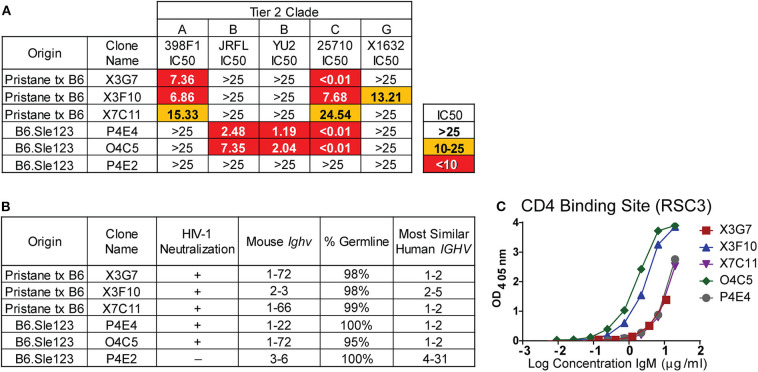
H2A-reactive mAbs that are also polyreactive neutralize HIV-1, are germline encoded, and express *Ighv* genes similar to human HIV bnAbs. **(A)** Purified H2A mAbs were tested for HIV neutralization using pseudotyped (JRFL, 25710, 398F1, and X1632) and infectious (YU2) viruses with TZMbl cells. IC50 was calculated as concentration of antibody needed for 50% neutralization. **(B)** Mouse *Ighv* genes were amplified from respective hybridomas using degenerate primers and sequenced. NCBI IgBlast was used to determine mouse *Ighv* genes, mutational frequency and homologous human *IGHV* genes. **(C)** 10 μg/ml of purified antibody from HIV neutralizing H2A-reactive mAbs were tested for reactivity against resurfaced stabilized core 3 (RSC3) CD4 binding site protein.

The global HIV-1 epidemic is dominated by seven viral subtypes or clades (40). Reference strains for these subtypes have been identified to test the breadth of antibody neutralization (40). These strains have been divided into three tiers based on resistance to neutralization (41, 42). To evaluate the breadth of neutralization from our previously identified (23) H2A-reactive clones from B6.Sle123 mice (Figure S2) and determine if the newly identified H2A-reactive mAbs from pristane-treated mice also neutralize HIV-1, we used a standard *in vitro* TZMbl assay to test the neutralization of five tier 2 strains from viral clades A, B, C, and G. Clone P4E2 was previously isolated from a B6.Sle123 mouse (23) and was included as a negative control mAb able to recognize H2A, but unable to neutralize HIV-1 (Figure S2 and Figure 2A). The results from these analyses revealed that all three of the H2A mAbs isolated from pristane-treated and gp140 immunized mice were also polyreactive (X3G7, X7C11, X3F10) and neutralized 2–3 tier 2 HIV-1 clades *in vitro* (clades A, C and G; Figure 2A). We further observed that the two IgM H2A mAbs previously isolated from gp140-immunized B6.Sle123 mice (P4E4, O4C5) (23) not only neutralize tier 2 clade B HIV-1 but also potently neutralized tier 2 clade C HIV-1 (Figure 2A). Interestingly, the neutralizing H2A mAbs derived from pristane-treated wild type mice were the only mAbs that neutralized both clade A and G viruses. These results demonstrate that H2A mAbs derived from two gp140-immunized mouse models of autoimmunity display some breadth in neutralization against tier 2 HIV-1 *in vitro*.

To further characterize our H2A mAbs, immunoglobulin heavy-chain variable (Ighv) regions were sequenced and analyzed for *Ighv* gene segment usage and degree of somatic hypermutation. We found that all but one of the neutralizing H2A mAbs used *Ighv* genes from the *Ighv1* family (1–72 and 1–22), while the non-neutralizing H2A-reactive mAb, P4E2, used *Ighv3-6* (Figure 2B). These data suggest that, similar to that observed in human HIV-1 bnAbs, there appears to be an *Ighv* gene preference used by the H2A-reactive mAbs that facilitates HIV-1 neutralization.

Human HIV-1 broadly neutralizing antibodies bear a high mutational frequency and show evidence of undergoing multiple rounds of somatic hypermutation (43). As such, we evaluated the degree of somatic hypermutation within our H2A mAbs and found they were 95–100% germline encoded (Figure 2B). These results show that H2A IgM mAbs from autoimmune prone mice can neutralize multiple clades of HIV-1 *in vitro* and do so with predominately germline encoded sequences.

A comparison of the mouse *Ighv* genes used by our H2A-reactive HIV-1 neutralizing antibodies to human *IGHV* genes showed that all but one of our HIV-1 neutralizing H2A mAbs were 75–79% similar to human germline *IGHV1-2* (Figure 2B) at the nucleotide level. Of note, human broadly bnAbs that recognize the conserved CD4 binding site (CD4bs) on the HIV-1 envelope protein gp120 are often encoded for by the human *IGHV1-2* gene (44). Accordingly, we assessed the reactivity toward the CD4bs epitope using the resurfaced stabilized core 3 (RSC3) CD4bs protein derived from the clade B HXB2 HIV-1 strain (45). This analysis demonstrated that all five HIV-1-neutralizing H2A mAbs also were able to recognize the CD4bs, albeit this reactivity was variable (Figure 2C).

### H2A-Reactive B Cells Are Present in the Peripheral B Cell Populations of Wildtype Mice

**Figure 3:**
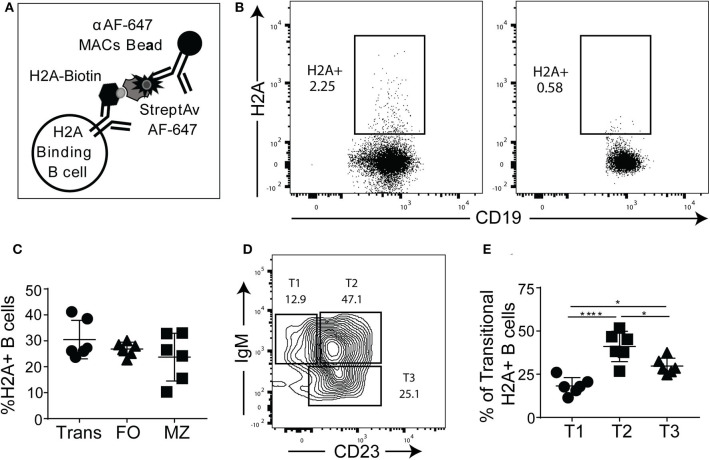
H2A-reactive B cells are present in wildtype mice. **(A)** Schematic describing the enrichment and identification of H2A-reactive B cells. **(B)** Representative analyses of viable splenic CD19^+^, singlet lymphocytes stained with H2A-biotin and streptavidin-Alexa Fluor 647, then enriched using magnetic beads reactive against Alexa Fluor 647 from wild-type C57BL/6 mice or BCR transgenic MD4 μMT mice. **(C)** Summary of frequency of H2A-reactive cells within transitional (CD24^hi^, CD23^+/**-**^ CD1d^lo/−^), mature follicular (FO: CD24^mid^, CD23^+^), and marginal zone (MZ: CD24^mid−hi^, CD1d^hi^, CD21^hi^) populations. **(D)** Representative flow plot for gating strategy used to define T1, T2, and T3 transitional populations (gated as CD24^hi^) based on IgM and CD23 expression. **(E)** Summary of the distribution of H2A-reactive B cells in the transitional populations T1 (CD24^hi^, CD1d ^lo/−^, IgM^+^, CD23^−^), T2 (CD24^hi^, CD1d ^lo/−^, IgM^+^, CD23^+^), and T3 (CD24^hi^, CD1d ^lo/−^, IgM^lo^, CD23^+^). Statistics: One-way ANOVA with Tukey's *post hoc* test. Two independent experiments with three mice each. *P ≤ 0.05; ****P < 0.00001.

Given that the H2A mAbs we isolated from autoimmune prone and pristane treated wild type mice were germline encoded, we next wanted to determine if H2A reactive B cells are present in naïve mice harboring wild type polyclonal repertoires. In these studies, we relied on recombinant human histone H2A that shares 98% amino acid sequence identity with murine H2A. To identify and enrich H2A-reactive B cells, H2A was conjugated to biotin and used to stain splenocytes, followed by staining with streptavidin-Alexa Fluor 647 and magnetic beads coupled to an anti-Alexa Fluor 647 mAb. These cells were then magnetically separated to positively enrich for H2A-binding B cells (Figure 3A), as previously described (10, 37). Importantly, Alexa Fluor 647 is a relatively small fluorochrome and thus presents significantly fewer epitopes able to be recognized by peripheral B cells within a wild type polyclonal repertoire compared to larger and proteinaceous fluorochromes such as phycoerythrin (data not shown). The results from these cell enrichments demonstrate the clear presence of H2A-reactive B cells in wild type mice. They also show that our enrichment was specific to the B cell receptor as the B cells from BCR transgenic μMT knock-out MD4 mice, which lack endogenous B cells and have a fixed specificity toward hen egg lysozyme, did not recognize H2A (Figure 3B). Following enrichment with magnetic columns, positive fractions were stained for markers that differentiate B cell subsets and analyzed by flow cytometry. Importantly, in the course of our studies we found that H2A histone non-specifically associates with apoptotic cells, possibly to exposed DNA on the surface of dying cells (Figure S3). Thus, to exclude dead cells from our analyses, dead cells were removed by Ficoll separation prior to cell staining, and strict live/dead gates were employed during data analysis.

Certain subsets of peripheral B cells, such as the transitional and marginal zone compartments, are known to harbor higher frequencies of autoreactive B cells (2, 46). Accordingly, we asked if H2A-reactive B cells, which recognize H2A as an autoantigen, were enriched in these populations. Our results demonstrated that the frequency of H2A-reactive B cells was similar across the transitional, follicular, and marginal zone populations (Figure 3C), similar to what has been observed for other endogenous antigen-specific autoreactive B cell populations (10, 47). We also analyzed H2A-reactive B cells for immunoglobulin kappa and lambda light chain expression and found normal distribution between the two populations (data not shown).

Autoreactive and anergic B cells are known to be enriched within the transitional B cell population, and specifically within the T3 subset (9). To determine if H2A-reactive transitional B cells were more prevalent within the T3 subset, we analyzed CD24-high B cells for CD23 and IgM expression to discriminate T1, T2, and T3 transitional B cell subsets (Figure 3D). We found that although the distribution of H2A-reactive B cells was higher in the T3 than in the T1 subset, the transitional population with the highest frequency was the T2 compartment (Figure 3E). This T2 subset is known to comprise the marginal zone B cell precursors (48). These results demonstrate that H2A-reactive B cells are present in naïve wildtype mice and distributed across all populations of B cells.

### H2A-Reactive B Cells Do Not Produce Autoantibodies or Respond to Immunization

**Figure 4:**
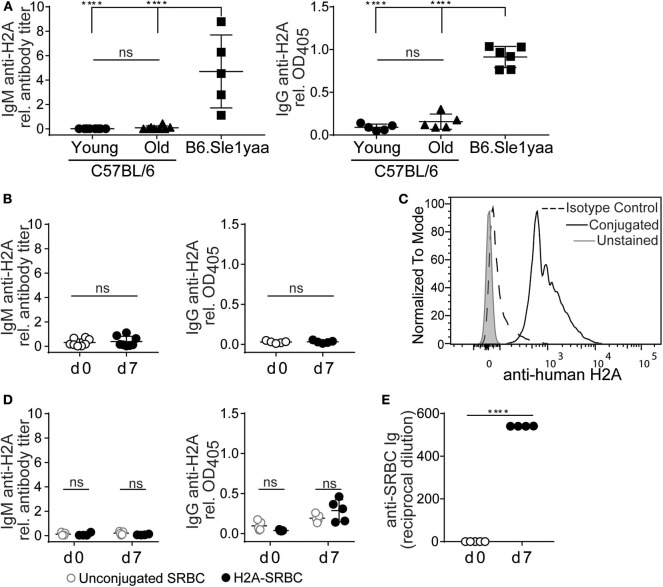
H2A reactive B cells do not produce antibody or respond to immunization. **(A)** Sera was collected from C57BL/6 young (8–13 wko) or older (5–7 mo) mice, and Sle1yaa mice (6 mo), and tested by ELISA for IgM (left) and IgG (right) reactive against H2A. Five to six mice per group. Statistics: One-way ANOVA with Tukey's *post hoc* test. **(B)** C57BL/6 mice were immunized intraperitoneally with 50 μg recombinant H2A in Alum. Sera was collected on day 0 and day 7, then tested for H2A-reactive IgM (left) or IgG (right). Results are cumulative of two independent experiments, three mice per group. Statistics: Student's unpaired *t*-test. **(C)** Representative histogram displaying H2A conjugation to sheep red blood cells (SRBCs). **(D)** 2 × 10^8^ unconjugated SRBCs (gray circles) or conjugated to recombinant H2A (black circles) were injected intravenously into C57BL/6 mice. Sera was collected on day 0 and day 7, and tested for H2A-reactive ELISA for IgM (left) or IgG (right). **(E)** Results for response to SRBCs, tested using hemagglutination assay. Two-way ANOVA with Tukey's *post hoc* test. Two independent experiments, 2–3 mice per group. Titers of autoantigen reactive IgM antibodies were calculated then normalized to EC50 values calculated from B6Sle123 serum sample positive control. For autoantigen reactive serum IgG, raw optical density values were normalized to optical density values obtained from B6Sle123 serum positive control at same dilution. ****P < 0.00001.

Autoreactive peripheral B cells in wild type mice have been characterized as being either functionally inert (anergic) or otherwise “ignorant,” but nevertheless do not secrete their autoantibodies *in vivo* (9). To assess whether endogenous H2A-reactive B cells were able to differentiate and secrete autoantibody we measured anti-H2A antibody in the sera of wild type young and older mice. Figure 4A shows that despite harboring H2A-reactive B cells, neither wild type young (8–13 weeks old) or older (5–7 months old) C57BL/6 mice harbored H2A-reactive IgM or IgG in their sera. In contrast, serum from a diseased male B6.Sle1yaa mice, which have a selective loss of tolerance against histone proteins (49), harbored easily detectable levels of both serum IgM and IgG H2A-reactive antibodies. These data suggest that H2A-reactive B cells do not spontaneously differentiate into antibody-producing cells *in vivo*.

To directly assess the ability of H2A-reactive B cells to respond to cognate autoantigen, we immunized wildtype C57BL/6 mice intraperitoneally with 50 μg of recombinant H2A with alum adjuvant. Serum was collected prior to immunization and 7 days post-immunization and analyzed by ELISA to detect H2A-reactive IgM and IgG. Following immunization with recombinant H2A, we found that wildtype mice did not produce detectable levels of serum H2A-reactive IgM or IgG (Figure 4B). This finding, together with the lack of H2A-reactive Ig in the serum of naïve mice, suggests that H2A-reactive B cells are not functional, and are likely silenced by peripheral immune tolerance.

It has previously been demonstrated that immunization with highly multimerized autoantigen can rescue autoreactive B cells from their functionally inert state and allow them to respond to self-antigen (30). Therefore, we next addressed whether a highly multimerized H2A antigen, i.e., presented on the surface of sheep red blood cells (SRBCs), could overcome the apparent functionally inert state of H2A-reactive B cells. We also reasoned that this approach could provide T cell help through linked recognition, by H2A-reactive B cells presenting antigens derived from SRBCs to CD4 helper T cells (50, 51). Thus, H2A was conjugated to SRBC and conjugation validated by flow cytometric analysis of SRBCs using polyclonal anti-human H2A IgG and compared to isotype control and unconjugated SRBC (Figure 4C). Wild type mice were injected intravenously with 2 × 10^8^ H2A-SRBCs or unconjugated SRBCs. Sera were collected prior to immunization and 7 days following immunization, then tested by ELISA for H2A Ig. The results from these analyses indicate that immunization with H2A-SRBC is also unable to elicit H2A-reactive IgM or IgG production, as compared to mice immunized with unconjugated SRBCs (Figure 4D). Importantly, mice immunized with H2A-SRBC did mount a robust antibody response against the SRBCs used for immunization as tested by hemagglutination assay (Figure 4E). These data indicate that, despite the absence of H2A-reactive antibodies, the route and method of inoculation used for the H2A-SRBC immunization was sufficient to generate an antibody response. Altogether, these results demonstrate that H2A-reactive B cells are functionally inert as measured by their inability to respond to immunization. These cells are also apparently unable to respond to highly multimerized antigen or receive help from wild type T cells.

### H2A-Reactive B Cells Do Not Respond to BCR Stimulation and Express Increased Levels of Inhibitory Mediators

**Figure 5:**
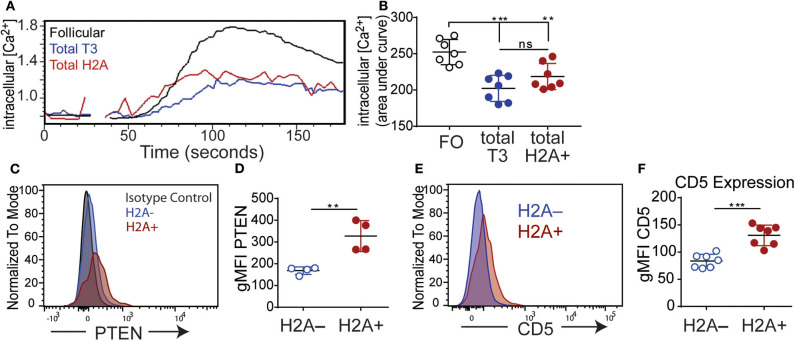
H2A reactive B cells display an impaired response to BCR stimulation and express markers of inhibition. **(A)** Kinetic representation of intracellular calcium concentrations in splenic Follicular (black), total T3 (blue) and H2A+ (red) B cell populations as indicated by Indo-AM1 dye following BCR stimulation with 10 μg/ml polyclonal anti-IgH&L Fab (Ab')_2_. Follicular B cell population defined as B220^+^ CD23^+^ CD24^mid^, and T3 population defined as B220^+^, CD24^+^, CD23^+^, IgM^lo^ **(B)** Summary of calcium mobilization data in Follicular, T3, and H2A reactive B cell populations, measured by area under the curve for Indo-AM1 ratio over time. Data are cumulative of two independent experiments, 3–4 mice per group. Statistics: One-way ANOVA with Tukey's *post hoc* test. **(C)** Representative histogram for PTEN expression in live singlet CD19+ H2A^+^ and H2A^−^ B cells. Isotype control depicted in gray dashed line. Two independent experiments, two mice per group. **(D)** Quantification of PTEN expression in live CD19^+^ H2A^−^ and H2A^+^ cells. Values displayed as geometric mean. Statistics: Student's unpaired *t*-test. **(E)** Representative histogram for CD5 expression in live singlet CD19^+^ H2A^+^ or H2A^−^ cells. **(F)** Quantification of CD5 expression. Values displayed as geometric mean. Two independent experiments, three mice per group. Statistics: Student's unpaired *t*-test. **P < 0.001; ***P < 0.0001.

A hallmark of anergy is the inability to efficiently transduce signals following a BCR stimulus. This can be assessed by measuring the magnitude of intracellular calcium mobilization following BCR stimulation. To directly measure if H2A-reactive B cells are functionally inert, splenocytes were labeled for markers defining B cell populations and stained with biotin-conjugated H2A. Importantly, in this assay H2A-reactive B cells were identified using an anti-biotin Fab fragment to avoid crosslinking BCRs with streptavidin and the premature initiation of BCR signaling. Cells were subsequently loaded with the calcium binding dye Indo 1-AM and stimulated with 10 μg/ml of polyclonal anti-IgM F(ab')_2_. Intracellular calcium levels were then monitored over time by flow cytometry. These results show that H2A-reactive B cells were clearly refractory to BCR stimulation as shown by a blunted calcium response, which was significantly decreased compared to follicular B cells while similar to that of the anergic T3 B cells population (Figures 5A,B). Importantly, analyses of calcium mobilization following stimulation with H2A directly conjugated to DyLight 650 showed that H2A alone was unable to induce intracellular calcium mobilization upon BCR signaling (Figure S4).

Several inhibitory phosphatases have been characterized to restrain BCR signaling and have been implicated in the inability of autoreactive anergic B cells to respond to BCR stimulation (52). Notably, levels of the phosphatase and tensin homolog (PTEN) phosphatase have been shown to be increased in both human and murine autoreactive anergic B cells (53, 54). Thus, we also evaluated the level of PTEN expression in H2A-reactive B cells (H2A+) vs. H2A-nonreactive (H2A-) B cells and found that H2A-reactive B cells indeed expressed higher amounts of PTEN compared to H2A-nonreactive B cells (Figures 5C,D). These results are consistent with our data demonstrating blunted calcium mobilization in H2A-reactive B cells, and further support the conclusion that H2A-reactive B cells are indeed functionally inert when examined immediately *ex vivo*.

We next assessed if H2A-reactive B cells express inhibitory surface molecules that could contribute to their inability to signal through the BCR. Previous findings have shown that autoreactive, anergic B cells express low levels of the surface molecule CD5 and genetic ablation of CD5 in anergic B cells leads to a loss of tolerance (55). As such, we analyzed the expression levels of CD5 on H2A-reactive B cells vs. H2A-nonreactive B cells and found that CD5 expression was also increased on the surface of H2A-reactive B cells and was similar for both Igκ and Igλ-positive H2A-reactive populations (Figures 5E,F; data not shown). H2A-reactive B cells did not display measurable increased expression of the CD72 or CD22 inhibitory receptors (data not shown).

In aggregate, the above data clearly demonstrate that endogenous wild type H2A-reactive B cells are functionally silenced by immune peripheral tolerance. Evidence to support this conclusion includes their inability to secrete autoantibodies under homeostatic conditions or after immunization, to inefficiently signal through the BCR, and by their increased expression of the PTEN phosphatase and the CD5 inhibitory receptor.

### TLR Stimulation or Autoimmune-Prone CD4 T Cell Help Breaks Tolerance in H2A-Reactive B Cells *in vitro*

**Figure 6:**
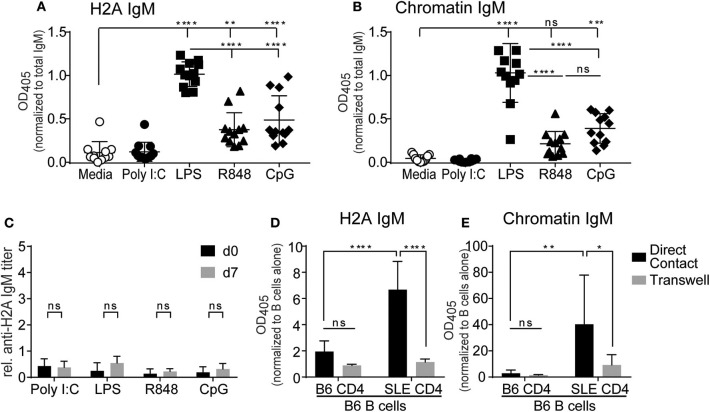
TLR stimulation and autoreactive CD4 T cell help breaks tolerance in H2A-reactive B cells *in vitro*. Splenic B cells from C57BL/6 mice were isolated and incubated with 20 ng/ml BAFF plus 10 μg/ml Poly I:C, Lipopolysaccharide, R848, or CpG ODN 1826. Antibody production was analyzed in culture supernatant after 10 days using an ELISA for **(A)** H2A and **(B)** Chromatin, then normalized to total IgM levels. Data represented by biological replicates. Three independent experiments. Statistics: One-way ANOVA with Tukey's *post hoc* test. **(C)** C57BL/6 mice were treated with either 100 μg of poly I:C, 50 μg of LPS, 100 μg of R848, or 100 μg of CpG ODN 1826. Serum was collected on day 0 and day 7 then tested for H2A-reactive IgM. Statistics: Two-way ANOVA with Tukey's *post hoc* test. Two experiments 3–4 mice per group. **(D)** CD4 T cells were purified and stimulated for 24 h with anti-CD28 and plate bound anti-CD3 in the presence of IL-2. CD4 T cells were then combined with purified B cells at a 1:1 ratio with 1.5 × 10^6^ cells of each cell type in 2 mLs total within a 24-well plate with or without separation by a 0.4 μm transwell. Samples were incubated for 8 days before culture supernatant was tested for autoantibodies by ELISA against H2A, or **(E)** Chromatin, then normalized to optical density values collected from respective samples incubated with B cells alone. Two independent experiments, 3–4 mice per group. Statistics: Two-way ANOVA with Tukey's *post hoc* test. *P ≤ 0.05; **P < 0.001; ***P < 0.0001; and ****P < 0.00001.

Despite their inability to respond to BCR stimulation, several studies have reported that autoreactive anergic B cells can produce antibodies following stimulation with TLR agonists (56–58). To determine if TLR stimulation could promote H2A-reactive B cells to produce autoantibodies, purified wild type splenic B cells were cultured *in vitro* with various TLR agonists together with the BAFF cytokine to promote cell survival and anti-H2A antibodies assessed in the secreted supernatant. TLR stimuli included poly I:C, LPS, R848, and CpG ODN 1826 for the stimulation of TLR3, TLR4, TLR7, and TLR9, respectively. B cells were cultured in the presence of stimuli for 10 days, culture supernatant collected and analyzed for IgM autoantibodies against H2A and chromatin. We found that when normalized to overall IgM concentrations in the supernatant, the production of IgM antibodies against H2A and chromatin were significantly increased in B cells cultured with agonists for TLR4, TLR7, and TLR9, with LPS (TLR4) stimulated cells producing the highest amount of antibodies against the various antigens tested (Figures 6A,B). In contrast, TLR3 stimulation using poly I:C did not induce production of either anti-H2A or anti-chromatin autoantibodies, which is consistent with previous findings demonstrating that TLR3 stimulation does not induce IgM production *in vitro* (59). These findings suggest that *in vitro* stimulation via certain TLRs can overcome the functionally inert state enforced upon H2A-reactive B cells *in vivo*.

We next wanted to evaluate the ability of TLR agonists to activate autoreactive H2A+ B cells *in vivo*. We reasoned that these experiments would be important as *in vitro* cultures with purified B cells would not account for the activation of other cells by TLR stimulation and would also be lacking in any tolerogenic cells that could inhibit autoreactive B cells. To do this, C57BL/6 mice were i.p. treated with either poly I:C, LPS, R848, or CpG ODN 1826. Blood was collected prior to treatment (d0) and seven days following treatment (d7) and analyzed by ELISA for the presence of H2A-reactive IgM. Anti-H2A IgM titers were calculated and normalized to a standardized stock of pooled autoimmune B6.Sle123 sera. As such, any major increase in autoantibody production would be indicated by a relative H2A IgM titer value of 1 or greater. Upon analysis of H2A-reactive IgM levels in TLR treated mice, we found no significant increase in autoantibodies against H2A (Figure 6C). These results suggest that tolerance continues to limit autoreactive B cells *in vivo* even in the presence of global TLR stimulation.

The results from our *in vitro* B cell cultures with TLR stimulus demonstrated that B cell mechanisms of intrinsic B cell tolerance can be bypassed with strong TLR stimulation. However, our experiments using *in vivo* TLR stimulation suggest the continued presence of B cell extrinsic tolerance mechanisms that restrict the activation of autoreactive B cells by TLR stimulation *in vivo*. Thus, we also assessed if CD4 T cell help was also able to promote the activation of autoreactive B cells from wild type mice. To accomplish this, we setup *in vitro* co-cultures with B cells from wildtype C57BL/6 mice and CD4 T cells from either wildtype C57BL/6 mice or autoimmune B6.Sle123 mice. B6.Sle123 are C57BL/6 congenic animals that carry three autoimmune-susceptibility loci from the autoimmune strain NZM2410. These mice also have known deficiencies in T cell tolerance and changes in activation of T cells (60, 61). We reasoned that autoreactive CD4 T cells from B6.Sle123 mice might feasibly provide the signals needed to activate H2A-autoreactive B cells and promote their production of autoantibodies. To further analyze this interaction and determine if any possible activation was cell contact-dependent or simply relied on cytokines, we cultured B cells and CD4 T cells together, or separated by a 0.4 μm transwell membrane. CD4 T cells were purified by negative selection from C57BL/6 and B6.Sle123 mice, activated *in vitro* with anti-CD3, and anti-CD28 in the presence of IL-2 for 24 h, and then cultured with purified C57BL/6 splenic B cells. Following an 8-day incubation, culture supernatants were collected and tested for antibodies against H2A and chromatin. We found that co-culture of C57BL/6 B cells with B6.Sle123 T cells resulted in the significant production of autoantibodies against H2A and chromatin (Figures 6D,E). In contrast, co-culture of wild type B cells with wild type T cells resulted in negligible autoantibody production (Figures 6D,E). The ability of B6.Sle123 T cells to promote autoantibody production was cell contact-dependent as C57BL/6 B cells cultured with B6.Sle123 CD4 T cells separated by transwell did not exhibit an increase in the production of IgM antibodies against H2A or chromatin (Figures 6D,E). Importantly, we did not detect significant autoantibody levels in the supernatant from purified CD4 T cells cultured alone (Figure S5), which discounts the possibility that the increased levels of autoantibody production by C57BL/6 B cells cultured with B6.Sle123 CD4 T cells was due to contaminating B cells in the B6.Sle123 CD4 T cell preparations. These findings, coupled with the results from our *in vitro* experiments with TLR stimulation, demonstrate that the functionally inert state displayed by H2A-reactive B cells is reversible and can be overcome *in vitro* by stimulation with TLR agonists or provision of CD4 T cell help.

### TLR Stimulation and Surrogate CD4 T Cell Help Breaks Tolerance in H2A-Reactive B Cells *in vivo*

**Figure 7:**
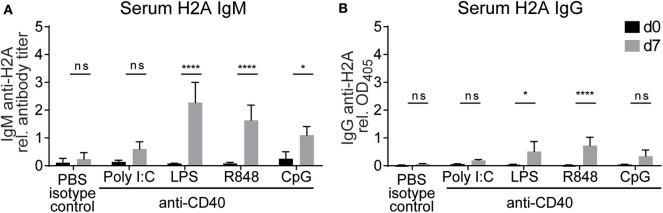
Treatment with anti-CD40 antibodies and TLR agonists induces IgM anti-H2A production *in vivo*. **(A)** C57BL/6 mice were treated with 50 μg of an agonistic antibody against CD40 (clone FGK45) in combination with either 100 μg of poly I:C, 50 μg of LPS, 100 μg of R848, or 100 μg of CpG ODN 1826. Controls were treated with PBS and rat IgG2a isotype control. Serum was collected on day 0 and day 7 then tested for H2A-reactive IgM and **(B)** IgG. Statistics: Two-way ANOVA with Tukey's *post hoc* test. Data representative of two independent experiments with 3–4 mice per group. *P ≤ 0.05; ****P < 0.00001.

Based on our previous *in vitro* experiments, which demonstrated that TLR stimulation and autoreactive CD4 T cell help can break tolerance in H2A-reactive B cells, we next wanted to evaluate the ability of CD4 T cell help and TLR stimulation to break tolerance *in vivo*. Because the activation of H2A-reactive B cells *in vitro* was contact dependent, we chose to substitute CD4 T cell help with anti-CD40 as a surrogate for the presumed co-stimulation provided by CD4 T cells through CD40 ligand expression. Wildtype C57BL/6 mice were injected intraperitoneally with anti-CD40 in combination with TLR agonists poly I:C, LPS, R848, or CpG ODN 1826. Sera was collected prior to treatment and 7 days following treatment, then analyzed by ELISA for H2A-reactive IgM and IgG. Similar to our *in vitro* results, we found that the production of antibodies against H2A was significantly increased in mice treated with anti-CD40 and agonists for either TLR4, TLR7, and TLR9 (Figures 7A,B). These results further support the finding that tolerance can be breached in H2A-reactive B cells by providing TLR stimulation and CD4 T cell help.

To determine if the increase levels of H2A-reactive antibody in serum allowed for the neutralization of HIV-1 *in vitro*, we tested serum antibodies from LPS anti-CD40 treated mice for neutralization against a panel of tier 1 and tier 2 clade B viruses. These analyses revealed that the anti-H2A autoantibodies elicited by *in vivo* treatment of wild type mice with LPS and anti-CD40 were not able to neutralize HIV-1 *in vitro* (Figure S6). These results suggest that the H2A-reactive antibodies elicited by LPS and anti-CD40 treatment *in vivo* do not originate from B cells able to generate a neutralizing response against HIV-1.

### H2A-Reactive mAbs From C57BL/6 Mice Display Minimal Polyreactivity and Poor HIV-1 Neutralizing Activity

**Figure 8:**
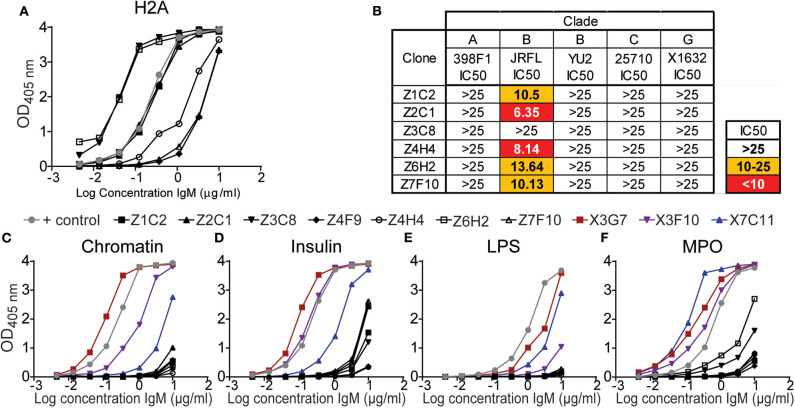
H2A-reactive mAbs from C57BL/6 mice display little polyreactivity and limited HIV-1 neutralization activity. **(A)** Purified IgM from hybridomas derived from C57BL/6 splenocytes treated with LPS and anti-CD40 (black data points) were tested for reactively against **(A)** H2A by ELISA starting at a concentration of 10 μg/ml. **(B)** 25 μg/ml of purified H2A mAbs were tested for HIV-1 neutralization using pseudotyped (JRFL, 25710, 398F1, and X1632) and infectious (YU2) viruses with TZMbl cells. IC50 was calculated as concentration of antibody needed for 50% neutralization. Purified IgM from hybridomas derived from C57BL/6 splenocytes treated with LPS and anti-CD40 (black data points) or HIV-1 neutralizing H2A-reactive mAbs derived from a pristane treated mouse (data points in color) were tested for reactively against **(C)** chromatin, **(D)** insulin, **(E)** lipopolysaccharide, and **(F)** myeloperoxidase by ELISA starting at a concentration of 10 μg/ml.

The results from above revealed that *in vivo* TLR stimulation together with surrogate T cell help (anti-CD40) significantly increased the production of serum H2A-reactive antibodies by wild type mice. We further determined that the highest levels of H2A-reactive IgM were elicited when anti-CD40 treatment was delivered in conjunction with LPS/TLR4 stimulation. However, the sera from these LPS/anti-CD40 treated wild type mice were unable to neutralize HIV-1. In contrast, we found that H2A-reactive sera elicited from either B6.Sle123 or pristane-treated C57BL/6 mice harbored HIV-1 neutralizing activity (Figure 2A and Figure S1). To explore features that correlate with the ability of H2A-reactive IgM antibodies to neutralize HIV-1, we generated anti-H2A IgM mAbs from wild type B cells treated *in vitro* for 48 h with anti-CD40 and LPS and tested these IgM mAbs for poly/autoreactivity. Sixteen H2A-reactive clones were originally identified of which six maintained H2A-reactivity following purification and analysis by ELISA at a standard concentration (Figure 8A).

The purified H2A-reactive mAbs derived from LPS and anti-CD40 treated C57BL/6 splenocytes were then tested for HIV-1 neutralization against a panel of multiple clades of tier 2 viral strains. We found that neutralization activity was limited to the tier 2 clade B virus JRFL (Figure 8B) whereas no neutralizing activity was observed toward other clades. These results suggest that the H2A-reactive mAbs generated from C57BL/6 mice have limited ability to neutralize HIV-1 and differ in some manner from the H2A mAbs generated from B6Sle123 and pristane treated mice.

We next assessed the polyreactivity of these newly generated H2A-reactive mAbs against chromatin, insulin, MPO, and LPS to test for reactivity against nuclear autoantigens, non-nuclear autoantigens, and a foreign antigen. We also included our poly/autoreactive HIV-1 neutralizing H2A-reactive mAbs from a pristane treated mouse for comparison and a reference level for polyreactivity. Following analysis by ELISA, we found that the mAbs derived from anti-CD40 LPS treated splenocytes displayed very little polyreactivity (black line data points, Figures 8C–F). This was opposed to what was seen with the mAbs derived from a pristane treated mouse, which were very polyreactive (data points in color Figures 1, 8C–F). Of note, although these anti-CD40 LPS derived mAbs recognize H2A, they did not display any reactivity for chromatin, which should presumably contain H2A or antigens similar to H2A. Therefore, these data demonstrate that our anti-CD40 LPS derived mAbs are largely restricted to the recognition of H2A and display limited polyreactivity. Thus, these data suggest that autoreactivity also associated with polyreactivity toward both self and non-self antigens correlate with a breadth of HIV-1 neutralization.

## Discussion

Immature B cells in the bone marrow assemble a B cell antigen receptor (BCR) via a stochastic process that relies on imprecision to generate BCRs with diverse specificities that also often include autoreactivity (2). Most of these autoreactive specificities are eliminated through central tolerance mechanisms that include receptor editing and clonal deletion. However, central tolerance is not absolute and B cells with certain (weak) autoreactive, and potentially pathogenic, specificities emigrate to peripheral lymphoid organs. In the periphery these autoreactive B cells are temporarily retained and restrained by mechanisms of peripheral tolerance. We argue that many of these autoreactive B cells whose BCRs also display polyreactivity may be retained to increase the diversity of the primary B cell repertoire and enhance the detection of pathogen epitopes. In this report, we identify and characterize within a wild type, polyclonal repertoire of B cells a population of histone H2A-specific autoreactive B cells with potential to neutralize HIV-1 but that are silenced by immune tolerance. The ability of immunological tolerance to limit the development of B cells that express a BCR with specificity for an HIV-1 neutralizing epitope has been demonstrated using immunoglobulin knock-in mice engineered to artificially express human HIV-1 bnAbs (19–22). The findings presented here thus extend our understanding of immunological tolerance as an obstacle for generating HIV-1 neutralizing antibodies within a wildtype, polyclonal and physiological system.

In this manuscript we confirm the previously established association between reactivity against the histone H2A autoantigen and the ability to neutralize HIV-1. Analysis of multiple H2A-reactive IgM mAbs isolated from wild type mice with pristane-induced autoimmunity show that these autoantibodies are often also polyreactive recognizing both self and foreign antigens. Further, using H2A-reactive IgM mAbs from two murine models of SLE, we demonstrated that H2A-reactive mAbs that are also polyreactive display a breadth of neutralization as evidenced by their ability to neutralize multiple subtypes of HIV-1 *in vitro*. Of note, the HIV-1 subtypes tested have been described as tier 2 pseudo-viruses. Tier 2 HIV-1 reference strains provide a good representation of circulating strains during infection, are more resistant to neutralization than tier 1 strains, and are considered relevant for evaluation of response to vaccination. Other studies have identified additional autoreactive antibody specificities toward double stranded DNA, phospholipids, and ubiquitin ligase 3, that when coupled to polyreactivity toward HIV-1 gp140 provide a breadth of neutralizing activity (62–64).

Sequence analysis of our H2A-reactive mAbs revealed that the HIV-1 neutralizing mAbs show preference for usage of Ig heavy-chain genes in the *Ighv1* family. This finding is of interest because the mouse *Ighv1* family shares the most sequence similarity with the human *IGHV1* family, a family whose gene segments are overrepresented in human bnAb clones that recognize the conserved CD4 binding site epitope on the HIV-1 envelope protein (44). Indeed, we found that our H2A-reactive mAbs also cross-reacted with a recombinant protein presenting the gp120 CD4 binding site. Although these results suggest that our mouse H2A-reactive mAbs display some similarities to human HIV-1 bnAbs, we also found that the mouse mAbs are nearly 100% germline encoded and polyreactive. To date, only one antibody, which was artificially engineered to express putative germline Ig heavy chain genes, has been described to display HIV-1 neutralizing activity (65). HIV-1 bnAbs isolated from human infants also display limited mutational frequency (66), but *de novo* germline HIV-1 neutralizing antibodies have not been described. As such, H2A-reactive B cells may represent a population of cells with fewer hurdles to overcome in order to contribute to a neutralizing response against HIV-1. Thus, our findings add support to several studies suggesting that a breach in immune tolerance could allow for a neutralizing antibody response against HIV-1 (21, 23, 26, 67, 68). It is important to note that the features of the HIV-1 neutralizing poly/autoreactive antibodies we identified, such as lack of somatic hypermutation and class-switch recombination, are typical of a short-lived antibody response. Therefore, questions remain about the durability of a poly/autoreactive humoral immunity. While this may influence the longevity of protection provided by the poly/autoreactive antibody response, it may also serve to protect the host from autoimmune manifestations.

Studies in BCR transgenic and Ig knock-in mice have demonstrated that B cells with an autoreactive specificity are limited by immune tolerance through mechanisms of receptor editing, clonal deletion and anergy (3, 4, 56, 69). These studies have laid the foundation for the understanding and analysis of B cell tolerance mechanisms, however; these models are also not physiological as they harbor an immune system with limited diversity in the primary B cell repertoire. Previous studies have also evaluated B cells in a wild type polyclonal repertoire reactive against autoantigens such as insulin, the RNA binding proteins La and SnRNP, glucose-6-phosphate isomerase (GPI), and the neo-self-antigen OVA (10, 70, 71). Results from these experiments have revealed that tolerance is not uniformly enforced on autoreactive B cells in a polyclonal repertoire and is dependent on factors such as the serum concentration of autoantigen and whether or not the autoantigen is soluble or membrane bound. Our data add upon these findings as we have identified *bona fide* endogenous H2A-reactive B cells within a polyclonal repertoire and characterized the effects of immune tolerance on this population.

We demonstrate that H2A-reactive B cells are functionally inert and subject to peripheral tolerance as indicated by their failure to respond to immunization *in vivo*, inability to respond to BCR stimulation *in vitro*, and expression of inhibitory mediators associated with the anergic state. It is of interest that despite their functionally inert state, H2A-reactive B cells are not overly represented in B cell populations known to harbor increased frequencies of autoreactive clones, such as marginal zone B cells. As such, we show autoreactive B cells can be distributed across all peripheral populations while being subject to mechanisms of both intrinsic and extrinsic B cell tolerance mechanisms.

Our findings also provide further confirmation that the peripheral restraints of immune tolerance can be at least in part breached by providing CD4 T cell help and strong TLR stimulation. This suggests that immune tolerance is a fragile and reversible state that can be transiently broken to allow for the activation of autoreactive clones under certain circumstances. Our findings corroborate previous studies that reported the activation of anergic B cells with TLR stimulation *in vitro* (56, 57). However, we found that TLR stimulation alone *in vivo* was not sufficient to drive the production of autoantibodies in wild type mice, suggesting that tolerance may continue to limit autoreactive B cells *in vivo* even in the presence of TLR stimulation. Importantly, the TLR stimulus provided may have been not strong enough to activate autoreactive B cells *in vivo*. It is important to note that the concentrations of each TLR agonist used in mice was considered to provide a “strong” stimulus. However, additional experiments to titrate the TLR treatments could provide further insight into the activation of H2A-reactive B cells by TLR stimulation alone *in vivo*. Finally, we found that the addition of T cell help-like stimuli simulated by an agonistic anti-CD40 antibody and in combination with TLR stimulation allowed for the production of H2A-reactive antibodies *in vivo*. Thus, our findings further elucidate the requirements for activating poly/autoreactive B cells, perhaps allowing them to contribute to a protective antibody response.

The quality and quantity of poly/autoantibody needed to be of use during a protective HIV-1 immune response remains to be elucidated, and the conditions that allow for the activation of poly/autoreactive B cells require further investigation. We showed that treatment with LPS and anti-CD40 increased H2A-reactive IgM to levels 2.5 times higher than what is observed in a standard stock of pooled autoimmune B6.Sle123 serum. However, when serum antibodies from LPS and anti-CD40 treated mice were tested for the ability to neutralize HIV-1, no neutralizing activity was observed. This suggests that the autoreactive H2A-binding clones activated with LPS and anti-CD40 may differ from the H2A-reactive clones activated in autoimmune-prone mice that can neutralize HIV-1. Notably, the H2A-reactive monoclonal antibodies generated from splenocytes treated with anti-CD40 and LPS displayed limited polyreactivity and HIV-1 neutralization breadth relative to those generated from autoimmune-prone mice. This anti-CD40 and LPS treatment was limited to splenocytes *ex vivo*, and thus only released autoreactive B cells from peripheral tolerance restraint. In contrast, the autoimmune-prone B6.Sle123 mouse strain has been characterized to harbor perturbations in both central and peripheral tolerance (61, 72, 73), and while it remains unclear how pristane-treatment of wild type mice leads to autoantibody production and autoimmunity, this treatment typically requires at least 1 month prior to autoantibody production during which time a breach in both central and peripheral tolerance could occur. Therefore, the differences in HIV-1 neutralizing ability observed between the H2A-reactive monoclonal antibodies derived from B6.Sle123 or pristane-treated mice vs. anti-CD40 and LPS treatment of wild type mice may reflect differences in the relaxation of central and/or peripheral tolerance checkpoints in these animals. Thus, suggesting that a compromise of both central and peripheral tolerance may be necessary to elicit a HIV-1 neutralizing response.

The “lock and key” model for antibody/antigen binding, which proposes that antibodies are monospecific for one antigen, has been the prevailing tenet in immunological thinking. While this is often true of a germinal center-experienced, somatically-mutated and affinity-matured antibody, the ability of a germline-encoded antibody to recognize structurally unrelated antigens, referred to as polyreactivity, has been a recognized feature of antibodies from the earliest analyses of myeloma proteins (74). Furthermore, analysis of multiple mAbs from both humans and mice suggest the presence of poly/autoreactivity in the B cell repertoire (2, 75, 76). Although many poly/autoreactive antibodies cross-react with self-antigen and could pose a potential autoimmune threat to the host, poly/autoreactive Ig, mainly IgM, and specifically natural IgM, is present in the serum of healthy individuals without obvious detriment (77). In fact, poly/autoreactive natural IgM has been implicated in the first line of defense against invading bacterium, clearance of apoptotic debris, and the maintenance of immune tolerance by enabling presentation of autoantigens to T cells in a tolerogenic manner (78–80). Although poly/autoreactive B cells have been shown to exist in peripheral B cell populations, studies by Wardemann et al. suggest that only 4% of mature naïve B cells possess a poly/autoreactive BCR (2). This frequency is reduced from 7% of “new emigrant” or transitional B cells, and even more so from immature B cells at 55% (2). While these studies used a limited panel of antigens to test for poly/autoreactivity, the results suggest that poly/autoreactive B cells are limited by immune tolerance. As such, the participation of poly/autoreactive B cells in a protective immune response may depend on a relaxation of B cell tolerance.

Our data support the idea that poly/autoreactivity is an advantage for the immune system as it increases the breadth of invading pathogens that the B cell repertoire could recognize. Furthermore, we believe that poly/autoreactive B cells may be at an advantage to recognize pathogens that may be masked by the molecular mimicry of self-antigen, such as HIV-1. This, together with findings that B cells can somatically mutate their specificity away from self and toward the minute differences in foreign sequence or structure (29–31), suggests that poly/autoreactive B cells represent a versatile population capable of formulating a protective response against a wide array of pathogens.

## Data Availability Statement

The original contributions presented in the study are included in the article/Supplementary Material, further inquiries can be directed to the corresponding author/s.

## Ethics Statement

The animal study was reviewed and approved by University of Colorado Anschutz Medical Campus Institute Animal Care and Use Committee.

## Author Contributions

RT and AA conceived the study, designed experiments, interpreted results, and wrote the manuscript. MS provided laboratory space, reagents and counsel for the HIV-1 neutralization experiments. RP contributed to experimental design, data interpretation, and manuscript editing. AA, JC, KS, and KG performed experiments included in this manuscript. All authors contributed to the article and approved the submitted version.

## Conflict of Interest

The authors declare that the research was conducted in the absence of any commercial or financial relationships that could be construed as a potential conflict of interest.

## Abbreviations

BCR: B cell receptor
HIV-1: Human immunodeficiency virus 1
bnAbs: Broadly neutralizing antibodies
MPER: Membrane proximal external region
mAb: Monoclonal antibody
TLR: Toll-like receptor
OVA: Ovalbumin
LPS: Lipopolysaccharide
BSA: Bovine serum albumin
Ighv: Immunoglobulin heavy chain gene
CD4bs: CD4 binding site
RSC3: Restabilized surface core 3
SRBCs: Sheep red blood cells
PTEN: Phosphatase and tensin homolog
SHP-1: SH2 domain containing protein tyrosine phosphatase
GPI: Glucose-6-phosphate isomerase.
