# Supplementary figures and images for: Histone H2A-Reactive B Cells Are Functionally Anergic in Healthy Mice With Potential to Provide Humoral Protection Against HIV-1
Source: Front Immunol. 2020 Jul 22;11:1565. doi: 10.3389/fimmu.2020.01565 (PMC7396680; doi:10.3389/fimmu.2020.01565)

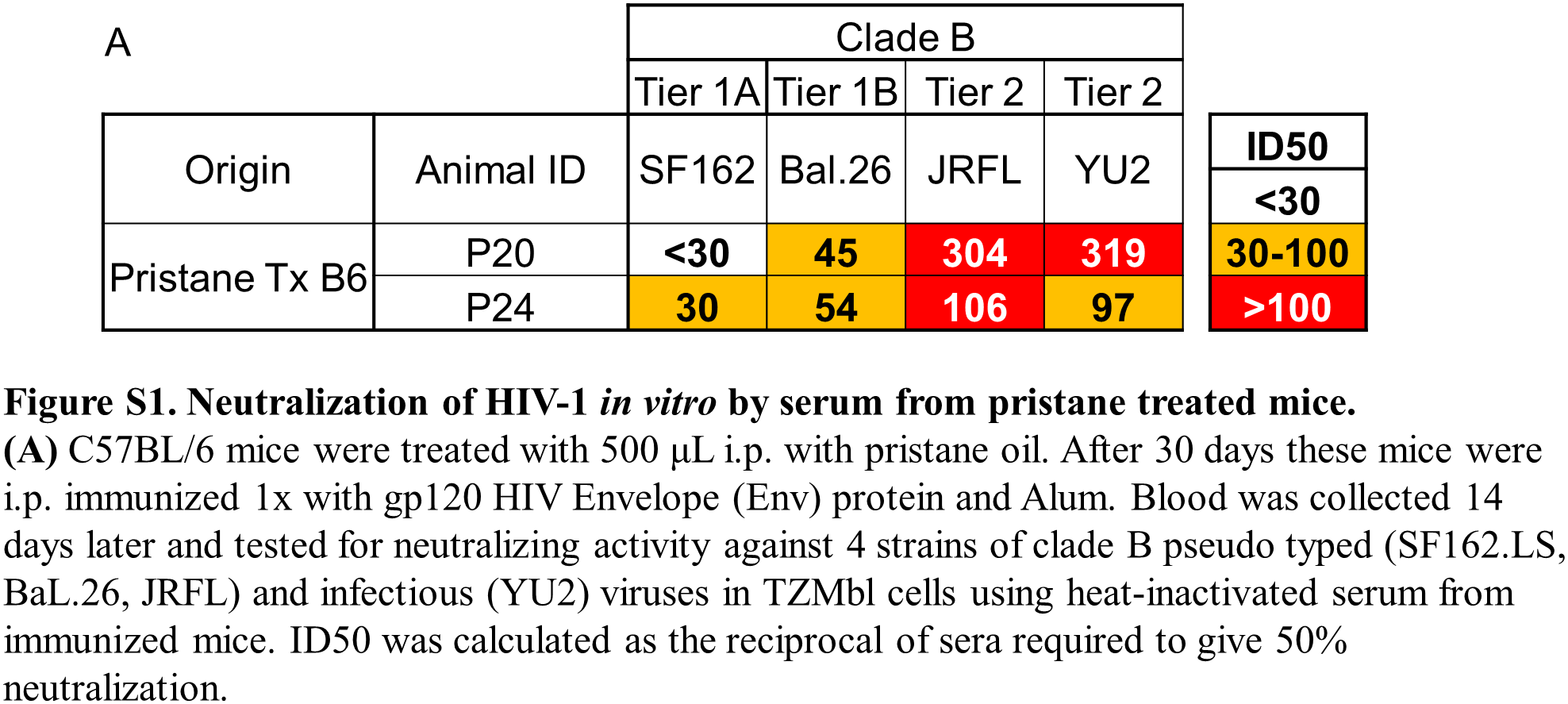

Supplement: Supplementary file 1 [file Image_1.TIF]

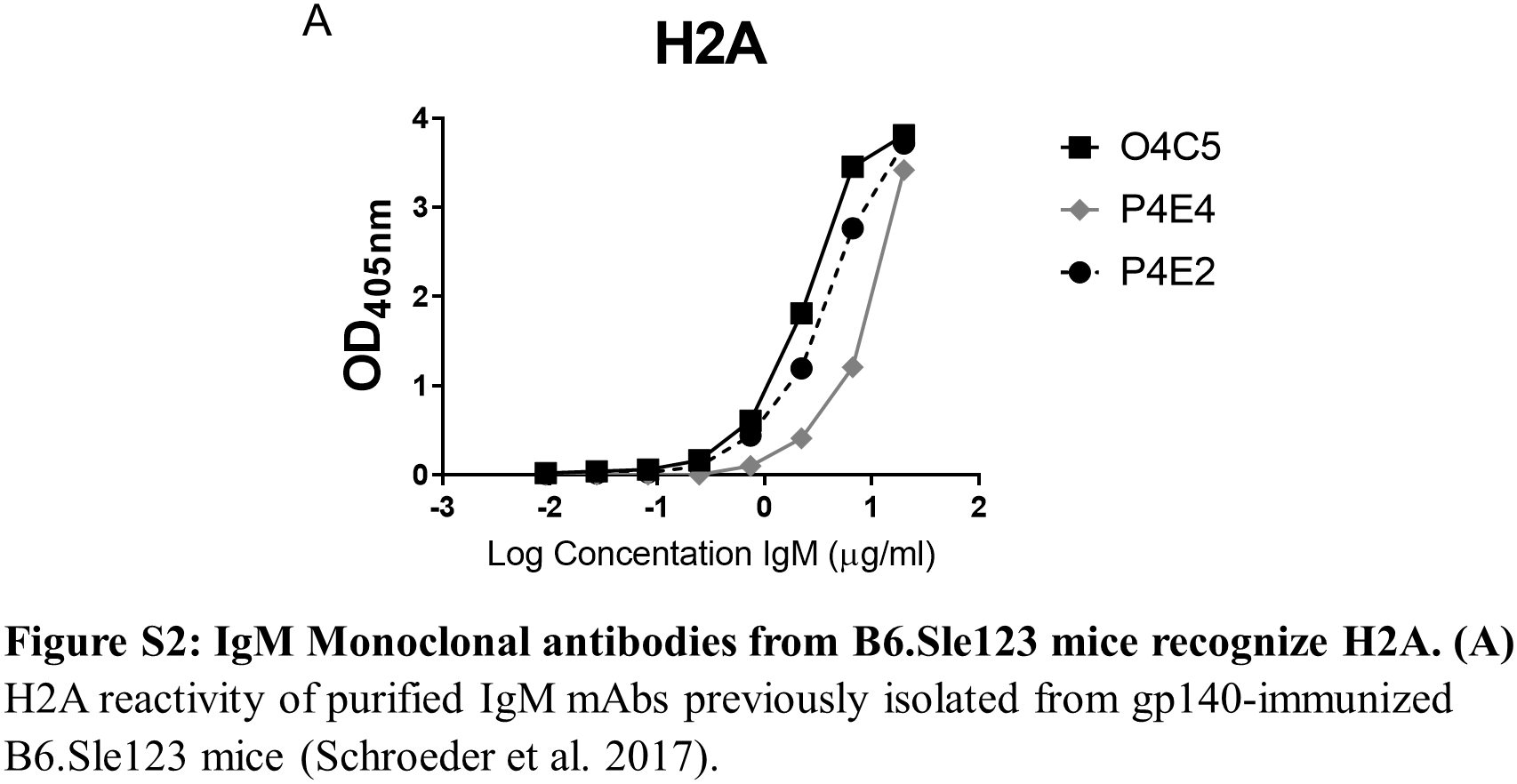

Supplement: Supplementary file 2 [file Image_2.TIF]

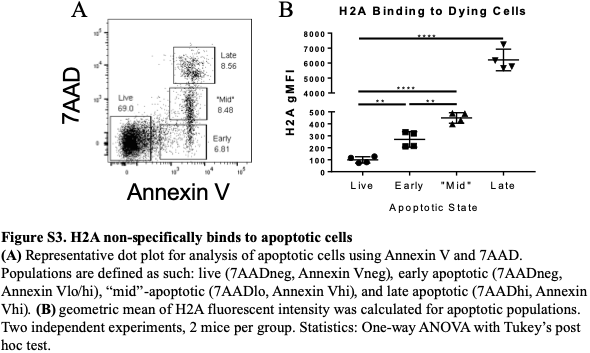

Supplement: Supplementary file 3 [file Image_3.tiff]

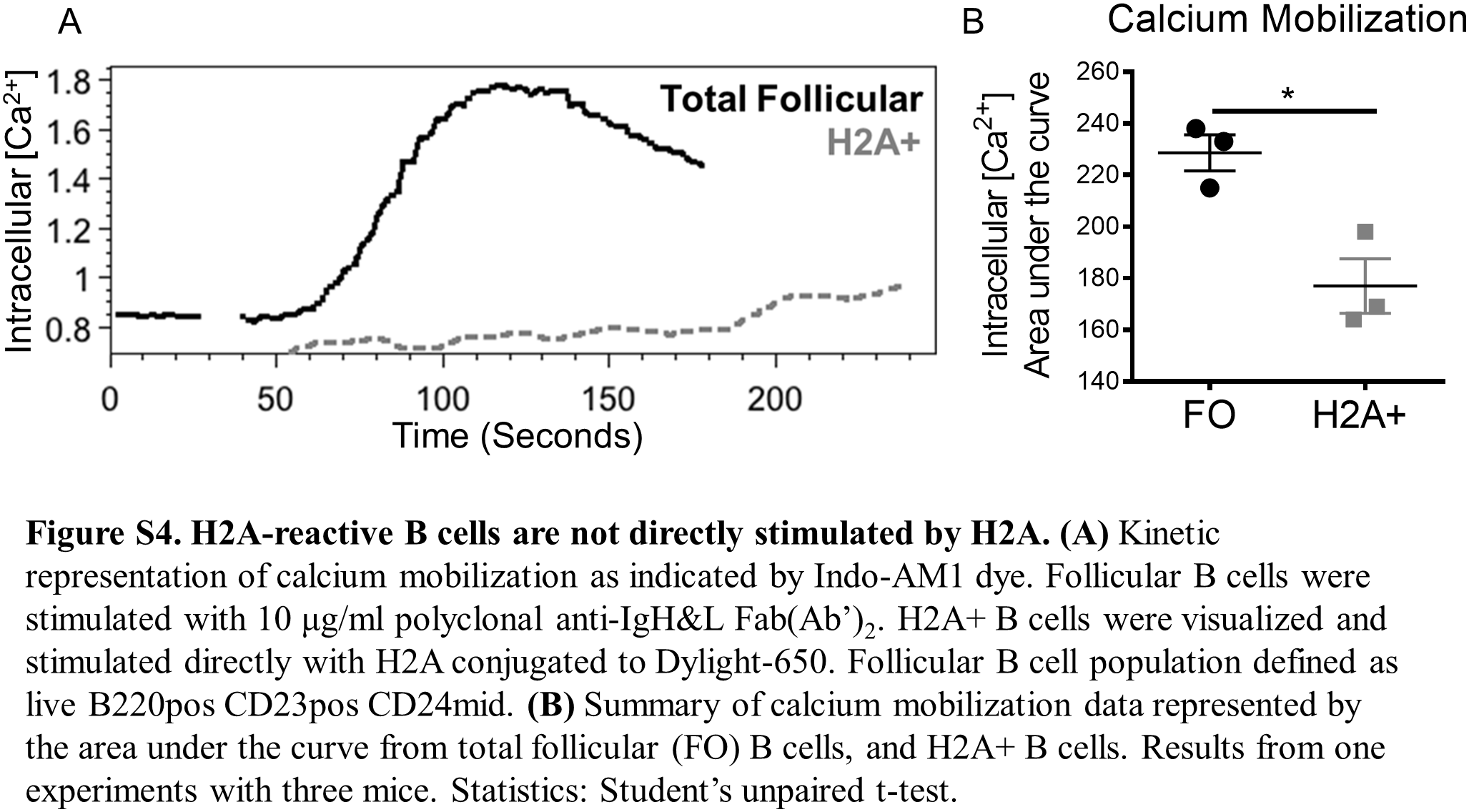

Supplement: Supplementary file 4 [file Image_4.TIF]

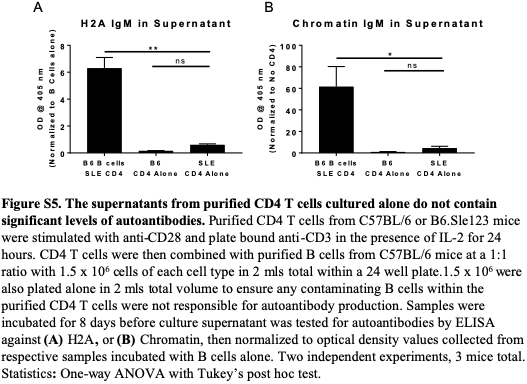

Supplement: Supplementary file 5 [file Image_5.TIFF]

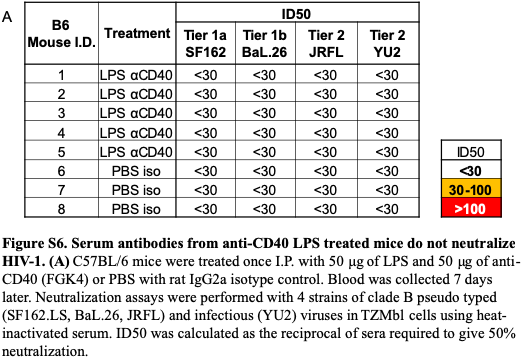

Supplement: Supplementary file 6 [file Image_6.TIFF]
